# Farming system shapes traits and composition of spider assemblages in Mediterranean cherry orchards

**DOI:** 10.7717/peerj.8856

**Published:** 2020-04-02

**Authors:** Natalia Rosas-Ramos, Laura Baños-Picón, José Tormos, Josep D. Asís

**Affiliations:** Departamento de Biología Animal, Facultad de Biología, Universidad de Salamanca, Salamanca, Spain

**Keywords:** Organic farming, Hillside aspect, Traditional orchards, Spider guilds, Cephalothorax width

## Abstract

Habitat properties, including crop type, farming system, management practices, or topographic features such as the hillside aspect, may act as environmental filters that select organisms sharing traits compatible with those conditions. The more environmentally-friendly management practices implemented in organic farming seem to benefit a range of taxa, but the extent of those benefits is not well understood. In cherry orchards of the Jerte Valley (Extremadura, western Spain), we explored the response of spider assemblages to the farming system (organic and conventional) and the hillside aspect (sunny or shady) from a taxonomical, behavioral, and morphological perspective. Spiders from both the canopy and soil surface were collected and identified to family. According to their foraging strategy, spiders were sorted in guilds and, for a selected family in each guild, body size was measured on each captured individual. Spider traits and composition were determined by local factors derived from farming system, and by climate conditions associated to the hillside aspect. In taxonomical terms, spiders benefit from organic farming and by the shady aspect. However, from a behavioral perspective, spiders with different foraging strategies exhibit strong variations in their response to the evaluated factors. From a morphological perspective, body size within guilds is differently conditioned by management practices that constitute conditioning disturbance events for each guild, resulting in selecting small individuals. The observed differences in taxonomical, behavioral, and morphological responses of spider communities to habitat properties highlight the importance of examining their assemblages from different perspectives when assessing how they respond to changes in management practices and topographic features.

## Introduction

Agriculture constitutes an important disturbance factor with strong impacts on the environment, and the current scenario of agrobiodiversity loss has elicited a growing concern about the sustainability of farming systems (Altieri, 1999; Tilman et al., 2002; Tscharntke et al., 2005; Feber et al., 2015; Lichtenberg et al., 2017; Sánchez-Bayo & Wyckhuys, 2019). To offset the negative impacts associated with the decline of biodiversity and its underlying ecosystem services, alternative farming approaches such as organic agriculture are being promoted (Hole et al., 2005; Rahmann, 2011; Froidevaux, Louboutin & Jones, 2017; Porcel et al., 2018). This low-intensity farming system, which implements environmentally-friendly management practices such as the ban of synthetic pesticides and fertilizers or techniques encouraging natural pest control, supports a higher biodiversity than conventional systems (Bengtsson, Ahnström & Weibull, 2005; Tuck et al., 2014; Feber et al., 2015; Froidevaux, Louboutin & Jones, 2017). Although the dichotomy between organic and conventional farming induces variations in community composition, the diversity of farming practices can also have an effect on biodiversity (Puech et al., 2014). Techniques similar to organic farming may be applied in conventional farms, whereas some practices that can be implemented in organic farms may not be especially environmentally friendly (Puech et al., 2014; Feber et al., 2015).

Several studies have emphasized that the effects of organic farming on biodiversity could be dependent on the landscape context and may vary across geographical regions due to differences in management practices or specific species composition (Bengtsson, Ahnström & Weibull, 2005; Tuck et al., 2014; Kehinde et al., 2018; Happe et al., 2019). Nonetheless, the effects of farming system on biodiversity have been investigated mainly in temperate regions (Froidevaux, Louboutin & Jones, 2017), and it is therefore necessary to assess their effects also in non-temperate agroecosystems, especially when they are located within a biodiversity hotspot for conservation priorities such as the Mediterranean basin (Myers et al., 2000; Hewitt, 2011). The effectiveness of organic farming can also vary among taxa, due to differences in resource exploitation strategies, niche preferences, or tolerance against disturbances (Bengtsson, Ahnström & Weibull, 2005; Holzschuh, Steffan-Dewenter & Tscharntke, 2010; Puech et al., 2014).

Certain habitat properties, including crop type, farming system, management practices, or topographic features such as the hillside aspect, may act as environmental filters that select organisms according to their physiological, morphological, and/or life-history traits (Schweiger et al., 2005; Podgaiski et al., 2013; Tuck et al., 2014; Xue et al., 2018).

When assessing community patterns, it is important not only to evaluate changes in community composition, but also to understand which morphological or ecological traits are selected against a certain disturbance (Gossner et al., 2015; Simons, Weisser & Gossner, 2016; Torma et al., 2019), since these characteristics can determine community resilience and may condition, among other outcomes, the effectiveness of natural enemies in biological control (Sunderland & Samu, 2000; Podgaiski et al., 2013).

Spiders are dominant invertebrate predators in many terrestrial ecosystems and occur in high abundance and richness in agricultural ecosystems, where they may play a role in the suppression of pest populations (Wise, 1993; Costello & Daane, 1998; Sunderland & Samu, 2000; Caprio et al., 2015; Drieu & Rusch, 2017; Michalko, Pekár & Entling, 2019). Selecting adequate indicators is essential in monitoring agroecological systems (Rosas-Ramos et al., 2019a) and, considering the sensitivity of spiders to ecological changes and human disturbances, this group of predators may constitute a promising bioindicator in assessing farming system effects (Marc, Canard & Ysnel, 1999; Gerlach, Samways & Pryke, 2013; Feber et al., 2015). Within a behavioral perspective, different spider guilds can be distinguished according to the similarities in foraging strategies: sheet, orb and space web-building spiders, stalking and ambushing spiders, and foliage and ground-running spiders (Uetz, Halaj & Cady, 1999). At a local scale, the occurrence of spiders can be determined, among other factors, by habitat structural features (e.g., vegetation structure, architectural complexity and heterogeneity), microclimate conditions, prey availability, or the occurrence of habitat disturbance events (Halaj, Ross & Moldenke, 1998; Heikkinen & MacMahon, 2004; Horváth et al., 2005; Entling et al., 2010; Spears & MacMahon, 2012; Podgaiski et al., 2013; Battirola et al., 2016; Gómez, Lohmiller & Joern, 2016). Habitat characteristics can drive spider assemblages in terms of taxonomy and life-history traits (Cardoso et al., 2011; Dennis et al., 2015), but may also determine morphological variations, all these traits affecting the structure and dynamics of food webs (De Souza & Martins, 2004; Woodward et al., 2005; Entling et al., 2010; Podgaiski et al., 2013; Rosas-Ramos et al., 2018; Michalko, Pekár & Entling, 2019).

In this study, we examine how farming system and hillside aspect, as a topographical feature, drive the assemblages of spiders from a taxonomical, behavioral (foraging strategy), and morphological (body size) perspective. We conducted the field study in sweet cherry orchards of western Spain, a major organic producer country. We first asked whether organic and conventional farming, as well as the aspect of the hillside in which the orchard is located (sunny or shady aspect) determine spider abundance and the number of spider families. It has been shown that organic fruit orchards seem to maintain a high overall biodiversity (Katayama et al., 2019; Happe et al., 2019), but responses could vary not only among taxa, but also depending on the evaluated traits. Morphological traits such as body size are determinant for spider adaptation and function in the environment and correlate with processes such as resource use, starvation, or desiccation resistance (Podgaiski et al., 2013). Since varying foraging strategies can result in spiders to be limited by different factors (Spears & MacMahon, 2012; Rosas-Ramos et al., 2018), we secondly asked how farming systems and the hillside aspect determine spider guild distribution and the individual body size within them.

## Material and Methods

### Study area

**Figure 1:**
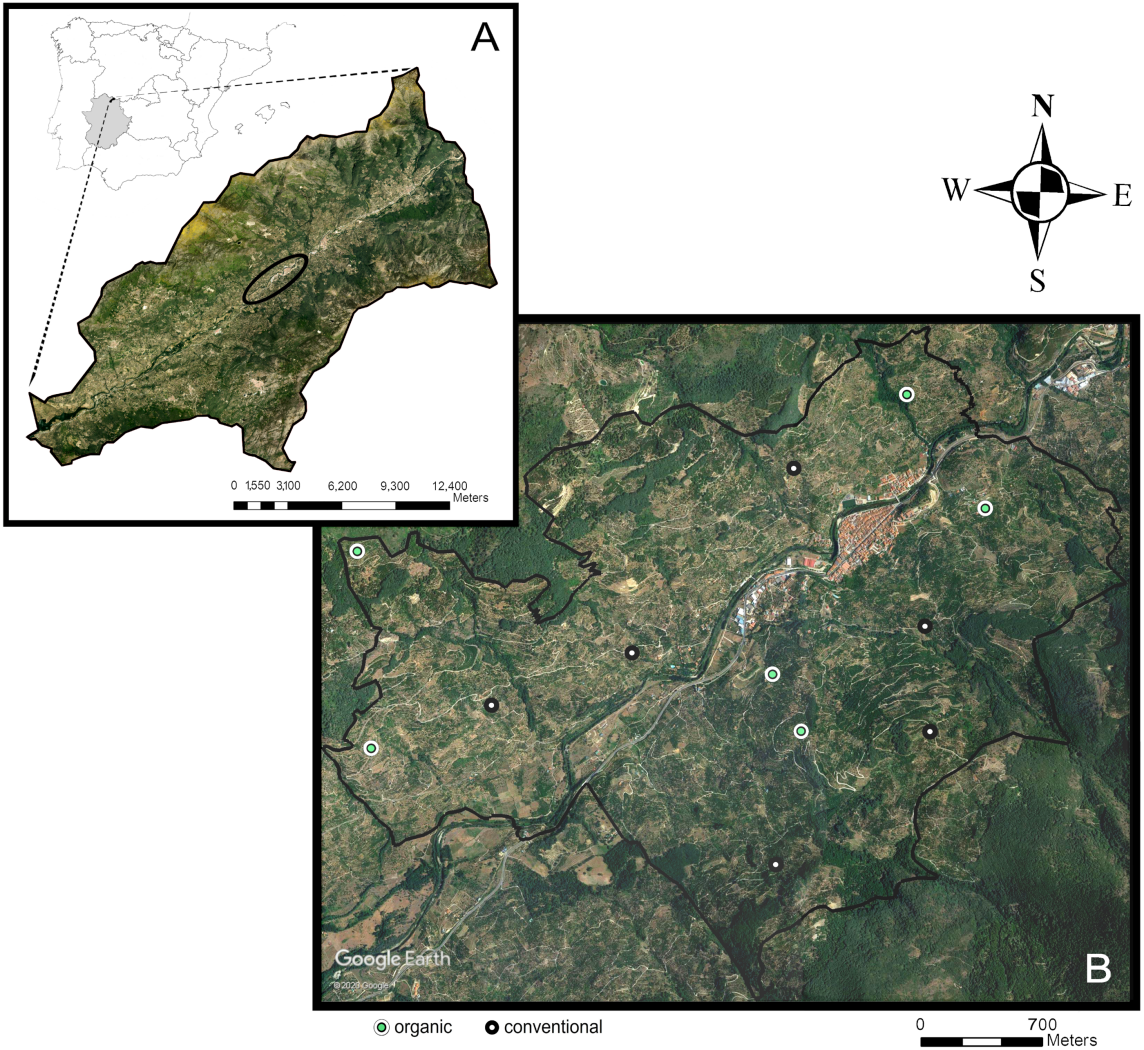
Study area. Location of the study area (black ellipse) in the Jerte Valley, in Extremadura, western Spain (A); location of the 12 sweet cherry orchards sampled across the study area (B) (obtained from Google Earth, 2020).

We conducted the study in the Navaconcejo municipality, in the Jerte Valley, an area located in the Spanish Central System mountain range (Extremadura, western Spain) (40°, 10′N, 5°50′W). The Jerte Valley runs linearly, with a northeast-southwest orientation, along the course of the Jerte River (Fig. 1). Bottom elevation ranges from about 360 m above sea level (a.s.l.) in the western end to 1,000 m a.s.l. in the eastern end. Hillside elevation also increases from west to east from 650 to 2,200 m a.s.l. The Valley has steep slopes (mean slope: 36%) and a higher solar radiation on the hillside with a southeast-facing exposition (sunny aspect) compared with the northwest-facing exposition (shady aspect). The narrowness, depth, and southern opening of the valley have an influence on its climate (Montalbán Pozas & Neila González, 2016), which is classified as Mediterranean continental mountain climate. It exhibits a marked seasonal contrast, having a warm period with high temperatures and low rain and a cold period with low temperatures and plentiful rain. Mean annual temperature ranges from 15 to 16 °C and mean annual rainfall from 1,000 to 1,100 mm, with August being the hottest and driest month (mean temperature: 26 °C; mean precipitation: nine mm).

The study area comprises 416.6 ha, dominated by traditional sweet cherry orchards (*Prunus avium* L.) with small and medium-sized fields (overall less than 1 ha) (Fig. 1), located within an elevation range of 500 to 950 m a.s.l. Orchards are composed of high-stem cherry trees cultivated on terraces separated by stone walls. Cherry production in the Jerte Valley is regulated by the Protected Designation of Origin “Cereza del Jerte” (D. O. P. Cereza del Jerte). The remaining natural vegetation is arranged according to an altitudinal succession, consisting of holm oak forests, oak forests, *Cytisus* shrublands, and alpine pastures. Additionally, the riverside vegetation is associated to the different gorges flowing into the Jerte river and includes alders (*Alnus glutinosa* L.), ashes (*Fraxinus angustifolia* Vahl), willows (*Salix alba* L.), poplars (*Populus* sp. L.), or birches (*Betula* sp. L.).

### Experimental design

Our field study comprised 12 conventionally and organically-managed cherry orchards located on both sunny and shady hillsides (three orchards for each of the four combinations: conventional-sunny, conventional-shady, organic-sunny, organic-shady) (mean plot area 6,414 ± 452 m^2^) (Fig. 1). Organic farmers manage cherry orchards according to the Council Regulation (2007), which is based on the ban of synthetic fertilizers and pesticides use. In organic orchards, the ground-cover consists of resident herbaceous vegetation punctually managed by mowing, whereas in conventional orchards, the ground cover vegetation is controlled by herbicide application, and the soil is mainly bare. Treatments in conventional orchards also include the application of synthetic fungicides (e.g., tebuconazol, difenoconazole, mancozeb, dodina), insecticides (e.g., lambda-cihalotrin, acetamiprid, tiacloprid, spinosad, cihalotrin, piroproxifen), and fertilizers (e.g., 4812, 91827, NPK).

### Spider sampling

We sampled the spider community of each of the 12 orchards monthly from April to August 2015 using two different methods (field study was approved by the Vicerrectorado de Investigación de la Universidad de Salamanca (USAL2015/18) and by the owners Francisco Acera, Roberto Díaz, Anibal Leralta, Jesús Carlos Manjón, Manuel Martín, Rafael Morales, Dionisio Moreno, José María Prieto, and Simeón Simón, who allowed us to do the samplings in their orchards). The sampling period in each month was selected avoiding atypical values of temperature or precipitation in order to ensure similar weather conditions. Canopy spiders were captured with a suction machine modified from a gardeners’ blower-vacuum (Avinent & Llácer, 1995). In each cherry orchard, we randomly selected 13 cherry trees that were vacuumed for 2 min along low, medium, and high strata. To collect spiders from the soil surface, we placed 20 pitfall traps (nine cm diameter, 12 cm depth) in each cherry orchard, arranged in linear transects along the terraces and separated by 12 m. Traps were filled with 100 ml of a solution of 70% alcohol and antifreeze/coolant (10%) in a 3:2 ratio (1:1 in August to avoid evaporation under the high temperatures); pitfalls remained active for four consecutive days each month. During spider sampling, geographic coordinates of every orchard were recorded. Spiders from both collecting methods were identified to family level (see Cardoso et al., 2011; Podgaiski et al., 2013). Several studies have confirmed the validity of using a higher taxon approach (e.g., family taxonomic resolution) to describe diversity patterns, and high correlations between data with high and low taxonomic resolutions have been found (De Oliveira et al., 2020).

### Spider guilds and body size

We assessed the response of spiders to farming system through a taxonomical approach and by using behavioral (foraging strategy) and morphological (body size) traits. According to their foraging strategy, we classified spiders into seven guilds, following the criteria by Uetz, Halaj & Cady (1999), Dias et al. (2010), and Cardoso et al. (2011): ambushers, stalkers, foliage runners, orb weavers, space web builders, sheet web builders, and ground runners. In addition, one family of each guild, among those families well represented in terms of abundance, was selected and each individual spider was measured. All individuals in these selected families were sorted to juveniles, females, and males, and we measured the cephalothorax width of adult females and juvenile spiders with a micrometer under a stereomicroscope, using this measure as a proxy of body size (Moya-Laraño et al., 2008). To perform the measurements, we selected the families Philodromidae (ambushers, 165 individuals measured), Salticidae (stalkers, 158 individuals measured), Anyphaenidae (foliage runners, 85 individuals measured), Araneidae (orb weavers, 47 individuals measured), Agelenidae (sheet web builders, 57 individuals measured), and Zodariidae (ground runners, 201 individuals measured). Space web builders were not considered since no family in the guild exhibited a sufficient representation.

### Data analysis

For data analysis, we pooled spiders from vacuuming and pitfall-trap samplings, since both methods exhibited high completeness values (non-parametric Chao 1 estimator (Colwell, 2009): 100% of the 14 and 26 estimated families for vacuuming and pitfall traps, respectively). We explored the spatial structure of spider assemblages by applying a Mantel test (Euclidean distance) between a matrix of geographic coordinates of sampled orchards and a matrix of Bray–Curtis similarity coefficients of spider family composition, finding no spatial autocorrelation (*r* = 0.1316, *p* = 0.204).

To analyze the differences in taxonomical composition of spider assemblages between organic and conventional cherry orchards, an analysis of similarities (ANOSIM) was applied. Similarities were calculated using Bray–Curtis coefficients, with a prior transformation of the original abundances (square root transformation). We conducted generalized linear models (GLMs) to evaluate the effects of the farming system (organic vs. conventional farming) and the hillside aspect (sunny or shady) on spider abundance and on the number of spider families. Models were tested for independence using the auto-correlation function (ACF) and, as no temporal correlation was found, sampling month was included in the models as a covariate. We analyzed the number of families using Poisson error structure and log link function, and abundance using negative binomial error structure to control for overdispersion. The optimal model was selected by stepwise backward selection.

The differences in spider guild composition between organic and conventional cherry orchards were evaluated using analysis of similarities (ANOSIM) (square root transformation; Bray–Curtis coefficients). Ordination of orchard types (organic-sunny aspect, conventional-sunny aspect, organic-shady aspect, and conventional-shady aspect) and spider guilds was carried out by Correspondence Analysis (CA) in order to represent the association between the abundances of each of the different guilds and the distinct types of cherry orchards evaluated (organic vs. conventional and sunny vs. shady hillside aspect placement). Additionally, generalized linear models (GLMs) were used to evaluate the effects of farming system (organic vs. conventional orchard management) and the hillside aspect (sunny or shady) on the abundances of such spider guilds. As a temporal correlation was not found, sampling month was also included in the models. Models were fitted with a Poisson distribution (log link function) or, when over-dispersed, with a negative binomial distribution. Optimal models were obtained by stepwise backward simplification.

Body size (cephalothorax width) variations within guilds under organic and conventional managements and in sunny and shady aspect were estimated by fitting a generalized least squares (GLS) regression model using restricted maximum likelihood estimation (Zuur et al., 2009). To deal with violation of independence, an autoregressive moving average model (ARMA (4,0) structure) was fitted to the errors in the residuals of the GLS model. To meet the assumption of homoscedasticity, the guild and the stage were included in the model as variance covariates. The optimal model was obtained by stepwise backward simplification.

The Mantel test was performed using the statistical program Past (Hammer, Harper & Ryan, 2001). We performed analyses of similarities (ANOSIM) in PRIMER 6.0 (PRIMER-E Ltd) (Clarke & Gorley, 2006). Generalized linear models and generalized least squares models were fitted with R 3.5.1 software (R Development Core Team, 2016). Correspondence Analysis was performed using XlStat, 2016 (Addinsoft, 2016).

## Results

### Spider abundances: families and guilds

We collected a total of 7,768 individuals belonging to 31 spider families (Data S1). Among these, 1,256 individuals were ambushers (six families), 596 were stalkers (three families), 271 foliage runners (five families), 105 orb weavers (two families), 96 space web builders (four families), 460 sheet web builders (five families), and 4,984 ground runners (six families).

### Taxonomical composition

**Figure 2:**
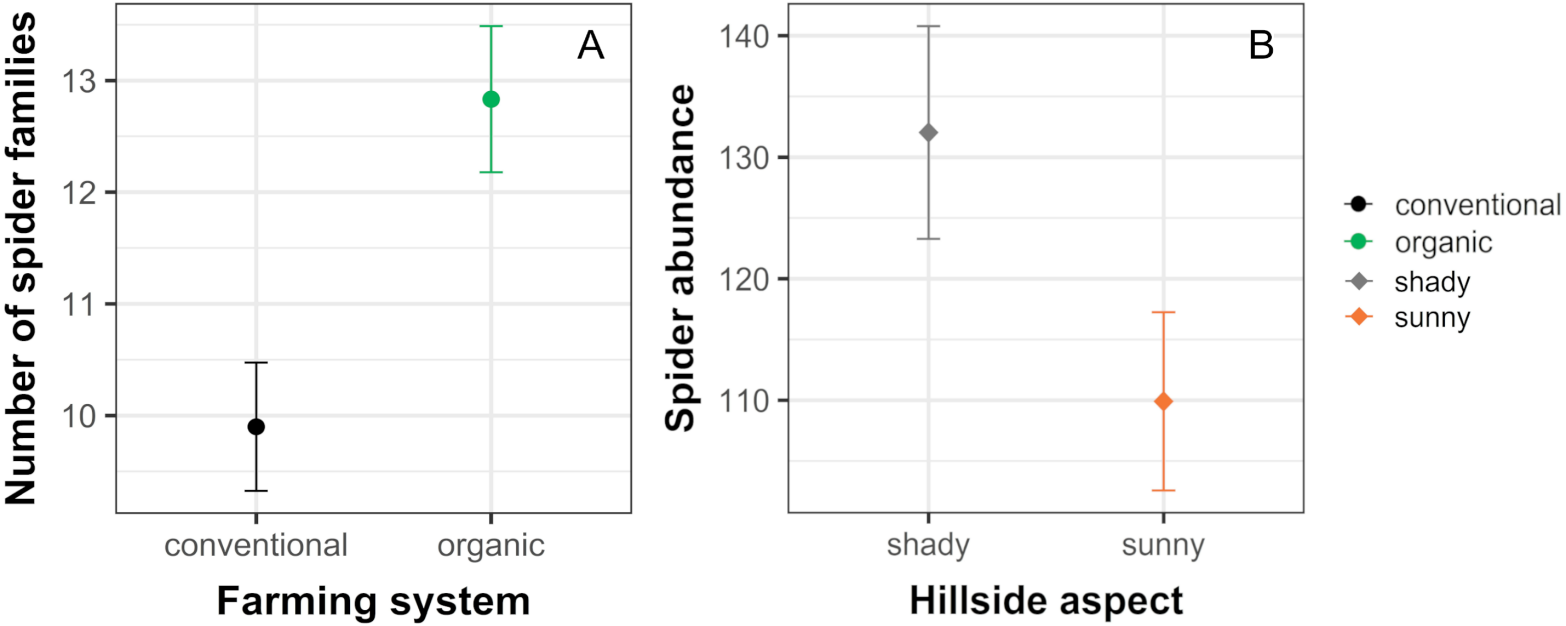
Significant effects of farming system and hillside aspect on spider abundance and the number of spider families. Estimated mean ± SE of (A) the number of spider families and (B) spider abundance in conventional and organic cherry orchards and in orchards from shady and sunny hillside. Parameters are estimated with a 95% confidence interval. Note that the scales of the vertical axes differ among graphs.

**Figure 3:**
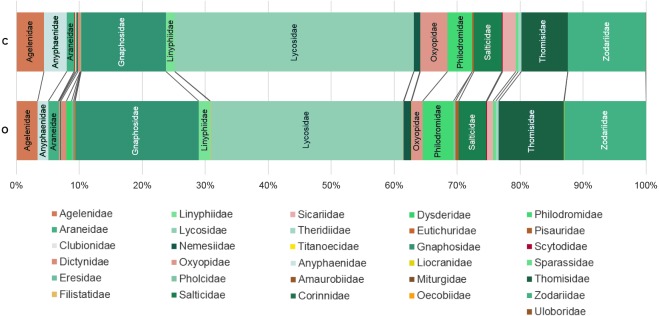
Percentage of individuals belonging to each family in each farming system. C: Conventionally managed cherry orchards; O: organically managed orchards.

The analysis of similarity (ANOSIM) revealed that taxonomical composition differed between organic and conventional cherry orchards (Global test: *R* = 0.126; *p* = 0.007). Results from the GLM showed that the number of spider families was significantly affected by farming system, but not by hillside aspect. Organically cultivated cherry orchards harbored richer spider communities compared with those conventionally managed (Table 1, Figs. 2 and 3). In terms of abundance, spiders were affected only by the hillside aspect, reaching the highest abundance values in cherry orchards located in the shady hillside (Table 1, Fig. 2). We found no significant relationship between spider abundance and farming system.

**Table 1:** Summary of the results of the spider taxonomical response to the farming system and the hillside aspect. Parameter estimates for the final generalized linear models (GLM) assessing the effect of the farming system (organic vs. conventional), the hillside aspect (sunny or shady), and the temporality (sampling month) on spider abundance and on the number of spider families. Parameters are estimated with a 95% confidence interval. Only significant variables are shown. Reference coefficients are system (conventional), aspect (shady), and month (April) (. <0.1; ^∗∗∗^*p* < 0.001).

**Response variable**	**Explanatory variable**	**Value**	**Std. Error**	*z*-Value	*P*	
*Number of spider families*	Intercept	2.292	0.058	39.510	<2e−16	***
	System (organic)	0.259	0.077	3.360	0.000779	***
*Spider abundance*	Intercept	4.709	0.115	40.785	<2e−16	***
	Aspect (sunny)	−0.183	0.094	−1.951	0.0511	⋅
	Month (May)	0.842	0.148	5.702	1.18e−08	***
	Month (June)	−0.045	0.149	−0.303	0.7621	
	Month (July)	0.142	0.149	0.956	0.3389	
	Month (August)	−0.069	0.149	−0.461	0.6445	

### Guild distribution

The analysis of similarity (ANOSIM) showed differences in guild composition between organic and conventional cherry orchards (Global test: *R* = 0.163; *p* = 0.001).

**Figure 4:**
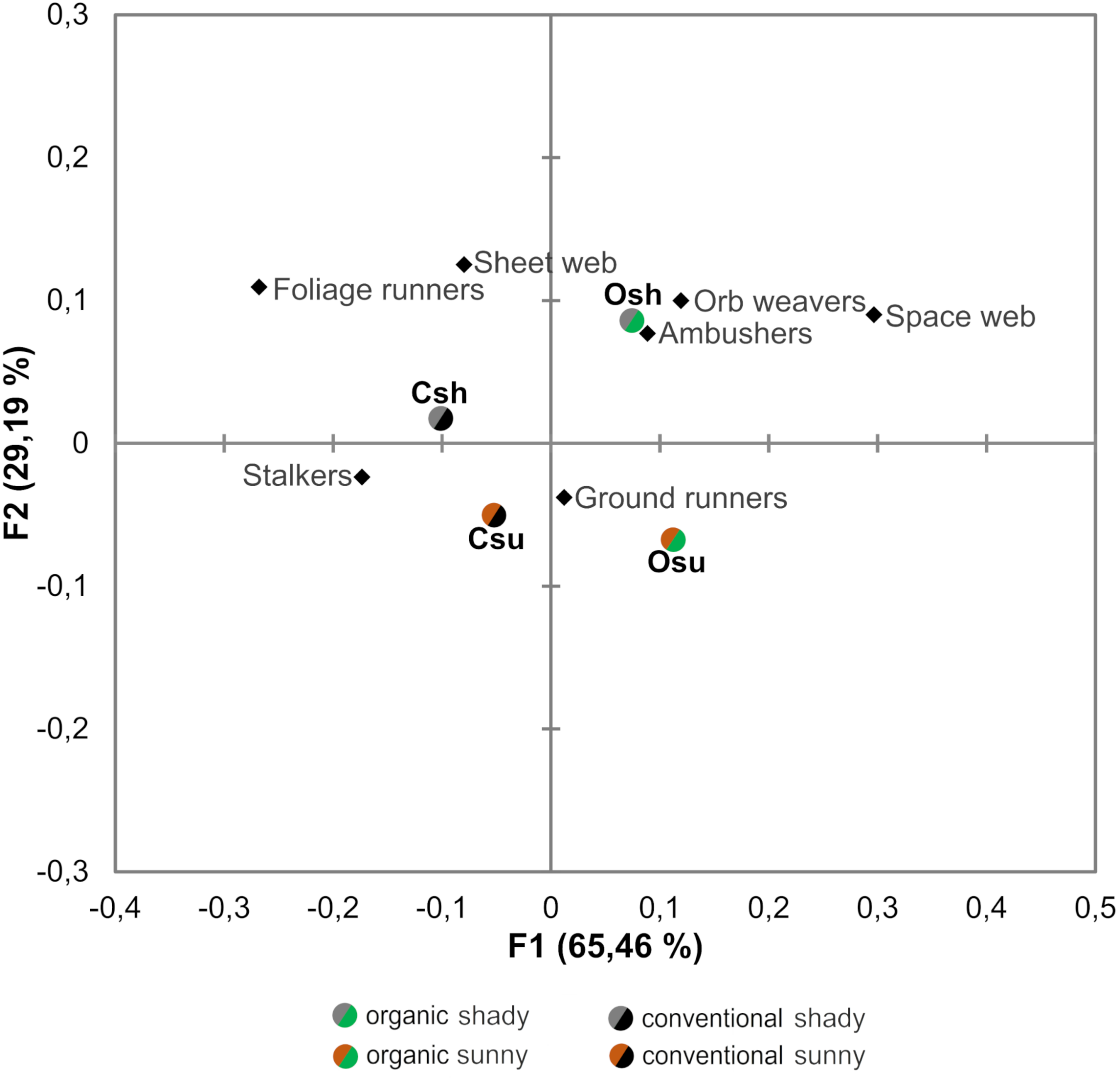
Correspondence analysis performed on the abundance of the different guilds of spiders associated to each orchard type. Osu: Organic cherry orchard located on the sunny hillside; Osh: organic orchard from the shady hillside; Csu: conventional orchard from the sunny hillside; Csh: conventional orchard from the shady hillside.

When evaluating the effects of farming system and hillside aspect on abundances within spider guilds, results from both the Correspondence analysis and GLMs revealed that the effects of such factors differ across guilds (Table 2, Fig. 4)

**Figure 5:**
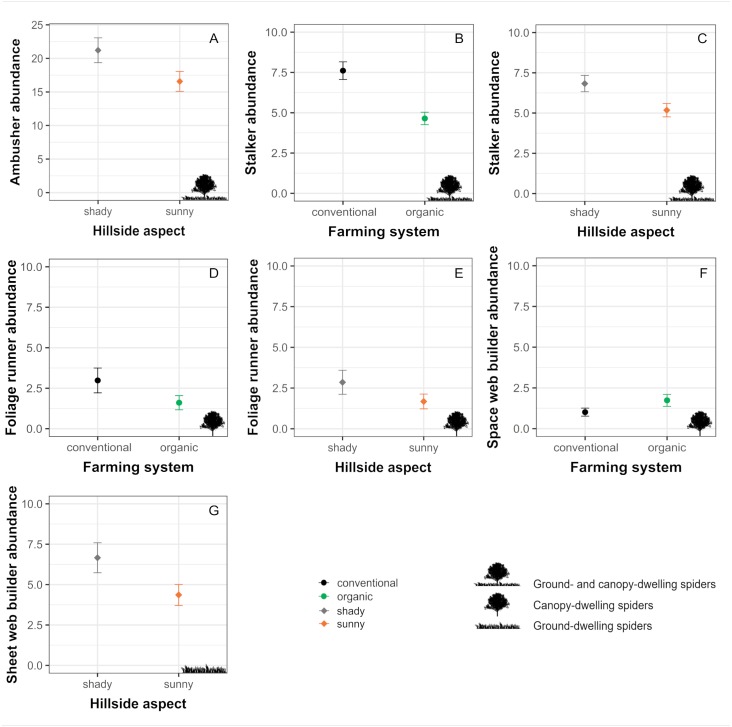
Significant effects of farming system and hillside aspect on the abundances of the different guilds of spiders. Estimated means ± SE of abundances in conventional (black dots) and organic (green dots) cherry orchards as well as in orchards located in the sunny (orange dots) and shady (grey dots) hillside for each of the different spider guilds: (A) ambushers and (B, C) stalkers (associated with both canopy and herbaceous ground cover); (D, E) foliage runners and (F) space web builders (associated mainly with the canopy); and (G) sheet web builders (associated mainly with the herbaceous ground cover). Parameters are estimated with a 95% confidence interval. Note that the scales of the vertical axes differ among graphs.

Ambushers, although tend to associate more to organic orchards, did not exhibit a significant response to farming system (Table 2, Figs. 4 and 5) and were affected only by the hillside aspect, reaching higher numbers in orchards located in the shady hillside (Table 2, Figs. 4 and 5). On the other hand, stalker and foliage runner abundances were significantly determined by both the farming system and the hillside aspect, being benefited by conventional farming and exhibiting higher numbers in orchards from the shady hillside (Table 2, Figs. 4 and 5). Orb weaver spiders, in spite of exhibiting a slight preference for organic orchards, did not show a significant response to any of the evaluated factors (Table 2, Fig. 4). For their part, space web building spiders reached significantly higher abundances in organic orchards, whereas sheet web builders exhibited higher numbers in orchards located on the shady hillside (Table 2, Figs. 4 and 5). Concerning ground runners, even though they were not significantly conditioned by neither the farming system nor the hillside aspect, they tended to associate more to orchards located on the sunny hillside (Table 2, Fig. 4).

### Body size

**Figure 6:**
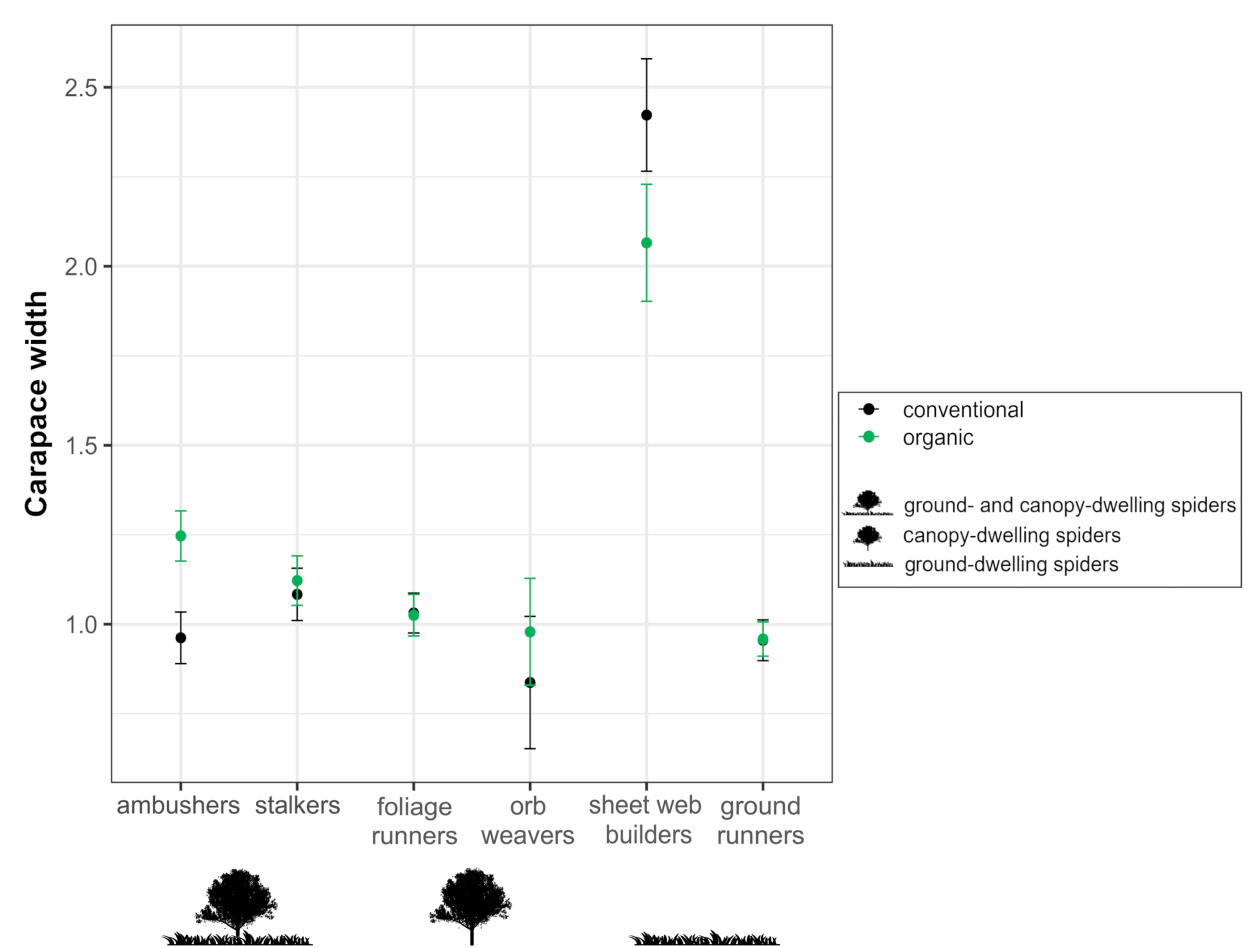
Effects of farming system on spider body size within guilds. Estimated mean ± SE of cephalothorax width in conventional (black dots) and organic (green dots) cherry orchards for each spider guild: ambushers and stalkers (associated with both canopy and herbaceous ground cover); foliage runners and space web builders (associated mainly with the canopy); and sheet web builders (associated mainly with the herbaceous ground cover). Parameters are estimated with a 95% confidence interval.

Regarding morphological traits, when analyzing the response of the spider individual body size (cephalothorax width) across guilds, the GLS showed a significant interactive effect between guild and farming system. The variation of spider body size in response to the farming system differed among guilds, both in adult females and juvenile spiders (Table 3, Fig. 6). Ambushers reached notably higher body sizes in organic than in conventional orchards, similarly than occurs, although to a lesser extent, with stalkers. On the contrary, sheet web builders responded to the farming system by exhibiting higher sizes in conventional orchards than in organic ones. Foliage runners and ground runners were significantly affected by farming system, but differences between organic and conventional orchards in the individual’s body size within each guild were scarce. Concerning orb weavers, no significant effect of the farming system on individual’s body size was detected.

**Table 2:** Summary of the results of the spider guild response to the farming system and the hillside aspect. Parameter estimates for the final generalized linear models (GLM) assessing the effect of the farming system (organic vs. conventional), the hillside aspect (sunny or shady), and the temporality (sampling month) on spider abundance of each guild. Parameters are estimated with a 95% confidence interval. Only significant variables are shown. Reference coefficients are system (conventional), aspect (shady), and month (April) (n. s. not-significant; . < 0.1; ^∗^*p* < 0.05; ^∗∗^*p* < 0.01; ^∗∗∗^*p* < 0.001).

**Response variable**	**Explanatory variable**	**Value**	**Std. Error**	*z*-Value	*P*	
*Ambusher abundance*	(Intercept)	2.425	0.163	14.853	< 2e−16	***
	Aspect (sunny)	−0.247	0.125	−1.981	0.0476	*
	Month (May)	1.202	0.201	5.983	2.19e−09	***
	Month (June)	0.186	0.212	0.879	0.3794	
	Month (July)	0.996	0.202	4.922	8.56e−07	***
	Month (August)	0.766	0.204	3.750	0.0002	***
*Stalker abundance*	(Intercept)	0.951	0.219	4.354	0.0000	***
	System (organic)	−0.493	0.084	−5.839	0.0000	***
	Aspect (sunny)	−0.277	0.083	−3.348	0.0008	***
	Month (May)	0.375	0.277	1.353	0.1761	
	Month (June)	0.952	0.251	3.793	0.0001	***
	Month (July)	2.589	0.221	11.712	< 2e−16	***
	Month (August)	2.166	0.225	9.625	< 2e−16	***
*Foliage runner abundance*	(Intercept)	−1.977	1.032	−1.917	0.0553	⋅
	System (organic)	−0.620	0.261	−2.378	0.0174	*
	Aspect (sunny)	−0.533	0.260	−2.047	0.0407	*
	Month (May)	3.866	1.059	3.651	0.0003	***
	Month (June)	4.325	1.055	4.100	0.0000	***
	Month (July)	4.392	1.055	4.165	0.0000	***
	Month (August)	4.096	1.057	3.876	0.0001	***
*Orb weaver abundance*	–	–	–	–	–	n. s.
*Space web builder abundance*	(Intercept)	−0.857	0.491	−1.747	0.0807	⋅
	System (organic)	0.539	0.313	1.719	0.0855	⋅
	Month (May)	0.690	0.582	1.185	0.2362	
	Month (June)	0.651	0.585	1.113	0.2656	
	Month (July)	1.573	0.544	2.891	0.0038	**
	Month (August)	1.438	0.548	2.624	0.0087	**
*Sheet web builder abundance*	(Intercept)	2.459	0.223	11.007	< 2e−16	***
	Aspect (sunny)	−0.423	0.199	−2.128	0.0333	*
	Month (May)	0.397	0.281	1.414	0.1573	
	Month (June)	0.043	0.285	0.152	0.8792	
	Month (July)	−1.498	0.335	−4.472	7.73e−06	***
	Month (August)	−1.755	0.352	−4.991	6.00e−07	***
*Ground runner abundance*	(Intercept)	4.323	0.159	27.132	< 2e−16	***
	Month (May)	0.868	0.224	3.877	0.0001	***
	Month (June)	−0.212	0.226	−0.939	0.3477	
	Month (July)	−0.383	0.227	−1.691	0.0908	⋅
	Month (August)	−0.455	0.227	−2.008	0.0446	*

## Discussion

Our study, performed in organic and conventional cherry orchards, highlights that the farming system shapes traits and composition of spider assemblages, as well as does the hillside aspect. We specifically show that spider community composition is determined both by local factors derived from the farming system (organic or conventional) and by climate conditions associated to certain topographic features (sunny or shady hillside aspect). Similarly, spider guild distribution and morphological features within guilds are mainly affected by farming system, followed by the hillside aspect, both factors shaping spider community traits.

**Table 3:** Summary of the results of the spider-size response to the farming system and the hillside aspect. Parameter estimates for the final generalized least squares model (GLS) assessing the effect of the farming system (organic vs. conventional), the hillside aspect (sunny or shady), the guild, and the stage (adult or juvenile) on spider size (cephalothorax width). Parameters are estimated with a 95% confidence interval. Only significant variables and interactions are shown. Reference coefficients are system (conventional), guild (ambushers), and stage (adults) (^∗^*p* < 0.05; ^∗∗^*p* < 0.01; ^∗∗∗^*p* < 0.001).

**Explanatory variable**	**Value**	**Std. Error**	*t*-value	*P*	
(Intercept)	1.287	0.085	15.127	0.000	***
System (organic)	0.285	0.073	3.891	0.000	***
Guild (stalkers)	0.122	0.095	1.284	0.200	
Guild (foliage runners)	0.070	0.092	0.753	0.451	
Guild (orb weavers)	−0.125	0.194	−0.642	0.521	
Guild(sheet web builders)	1.461	0.169	8.656	0.000	***
Guild (ground runners)	−0.007	0.087	−0.078	0.938	
Stage (juvenile)	−0.615	0.051	−12.147	0.000	***
System (organic): Guild (stalkers)	−0.246	0.104	−2.379	0.018	*
System (organic): Guild (foliage runners)	−0.291	0.091	−3.206	0.001	**
System (organic): Guild (orb weavers)	−0.143	0.164	−0.873	0.383	
System (organic): Guild(sheet web builders)	−0.641	0.157	−4.078	0.000	***
System (organic): Guild (ground runners)	−0.281	0.097	−2.896	0.004	**

### Taxonomical composition

Spiders are highly sensitive to environmental change, and their strong dependence on habitat structure is a key factor in shaping their assemblage composition (Topping & Lövei, 1997; Halaj, Ross & Moldenke, 1998; Marc, Canard & Ysnel, 1999; De Souza & Martins, 2005; Cardoso et al., 2011; Rosas-Ramos et al., 2019b). The maintenance of ground cover vegetation in organic orchards leads to a greater structural diversity, which involves ecological niche diversification and facilitates the coexistence of different groups of spiders. This is reflected by the presence of richer spider communities, as predicted by the habitat heterogeneity hypothesis (Tews et al., 2004; Jiménez-Valverde & Lobo, 2007; Podgaiski et al., 2013). Ground cover vegetation not only increases structural diversity, but also plant diversity, both factors that potentially enhance prey availability (Loomis & Cameron, 2014; Ebeling et al., 2018), which is a limiting factor for spiders (Halaj, Ross & Moldenke, 1998; Horváth et al., 2005; Markó et al., 2009; Spears & MacMahon, 2012; Loomis, Cameron & Uetz, 2014; Dennis et al., 2015) and accordingly, we would expect the organic orchards to support also higher numbers of spiders. Nevertheless, the effect of farming system was not significant in terms of abundance, maybe because the ability of this generalist predators to feed upon a wide range of prey through a variety of strategies (Wise, 1993; Marc & Canard, 1997) could be buffering the potential effect of farming system on spider abundance.

When evaluating the effect of the hillside aspect on spider community, we found that the number of spiders, but not their richness (number of families), increased under the microclimatic conditions offered by the shady aspect. It has been demonstrated that spiders are strongly conditioned by abiotic factors such as shading, moisture or temperature (Wise, 1993; Entling et al., 2007), the latter being determinant for spiders’ life history mechanisms including net casting, scape speed, feeding and growth, or the survival of juvenile stages (see Napiórkowska et al., 2018) which are determinant factors for spider abundance. The greater densities found in shady aspect might suggest that these orientation provides more favorable climatic conditions for spiders. Conversely, spider richness may be more conditioned by structural features such as vegetation complexity than by environmental factors such as temperature and humidity (Jiménez-Valverde & Lobo, 2007).

### Guild distribution and body size

The diversity in foraging strategies exhibited by spiders makes each guild constrained by different factors. Additionally, body size within guilds vary in response to management practices that constitute conditioning disturbance events for each group.

The tendency of ambusher spiders to associate more to organic orchards, where they exhibited significantly greater body sizes, can be due to the hunting strategy that displays this guild. It consists of attacking their prey from a close proximity, which makes them to be favored by the availability of more concealed locations for prey capture offered by structurally diverse environments (Hatley & Macmahon, 1980; Uetz, 1991; Spears & MacMahon, 2012). Thus, the greater local structural diversity exhibited by organic orchards, derived from the maintenance of ground cover vegetation, could be favoring this guild. In contrast, stalker spiders are active hunters that leap onto their prey and benefit by habitats with a more open structure, which facilitate their vision and jumping ability, making prey catching easier (Hatley & Macmahon, 1980; Robinson, 1981; Spears & MacMahon, 2012). This could be the reason why stalkers showed a preference for conventional orchards although exhibiting smaller body sizes, that might be derived from pesticide use in orchards under this management system. The application of pesticides selects smaller spiders, which are less sensible to such treatments since a smaller body size would collect fewer droplets after spray application, inducing lower mortality rates (Pekár, 2012).

Different studies have shown that the number of canopy spiders decreases with increasing pesticide use, due to direct or indirect effects of the treatments (Bogya, Markó & Szinetar, 2000; Markó et al., 2009). Therefore, we would expect foliage runners to be more associated to organic orchards. However, our results showed that organic orchards benefit foliage runners neither in terms of body size (Pekár, 2012) nor in terms of abundance, maybe because differences in structural features derived from the presence or absence of ground cover vegetation do not constrain canopy-dwelling spiders (Costello & Daane, 1998).

In our study, guild responses to organic and conventional farming in terms of abundance were less pronounced in web-building spiders compared to hunting spiders, suggesting that the former are less conditioned by farming system. Many authors have documented that sensitivity to pesticide use could depend on spider foraging strategy (Markó et al., 2009; Pekár, 2012). For instance, web-building spiders are more resistant to pesticides than hunting spiders because webs efficiently collect sprayed chemicals and protect the spiders from direct contact, thus making them less conditioned by the farming system (Pekár, 1999; Pekár, 2012). In this regard, orb weavers did not exhibit differences in body sizes between organic and conventional orchards, reflecting how pesticide treatments do not strongly constrict this guild.

Regarding sheet web building species, the configuration of their webs make these spiders to have more strict spacing tolerances than other weavers (Rypstra, 1983; Blamires, Zhang & Tso, 2017). These restrictions could explain why individuals from this guild reach higher body sizes in conventional orchards and why the farming system did not drive the response of sheet web-builders in terms of abundance; the obtained patters may reflect how punctual mowing can be mitigating the potential benefits of organic management and can counteract individuals from this guild.

The availability of a suitable microclimate or a reduced predation risk may determine microhabitat selection by ground runners (Bell, Philip Wheater & Rod Cullen, 2001; Rypstra et al., 2007) and thus, higher densities of these spiders would be expected in more complex habitats such as organic orchards. However, we found no effect of farming system on abundance, and only a slightly positive effect of organic farming on ground runner body size. This suggests that spiders from this guild benefit to some extent from the ground structure characterizing these organic orchards, but this positive effect would be scarce. Previous studies have demonstrated that some ground runner spiders prefer open habitats and bare soil (Bell, Philip Wheater & Rod Cullen, 2001; Moretti et al., 2002; Podgaiski et al., 2013; Rosas-Ramos et al., 2018), which could be buffering the potential benefits of organic farming comparing to conventional systems.

## Conclusions

Both farming system and topographic features act as environmental filters that shape spider assemblages not only in terms of taxonomy, but also regarding guild composition and morphological traits (body size) within each specific guild. Our findings support that, from a taxonomical approach, spiders show a consistent response, benefiting from organic farming and being also favored by the shady aspect. However, when evaluating the spider assemblage from a guild perspective, we obtained a more heterogeneous response because each guild is limited by specific requirements. Thus, spiders with different foraging strategies varied strongly in their responses to farming system, since each guild was limited by specific local features derived from management practices developed in organic and conventional regimes (e.g., mowing or pesticide and herbicide application). Our work also indicates that body size varies in response to management practices that constitute conditioning disturbance events for each guild, resulting in selecting smaller individuals within them. In the light of our results, we stress the importance of examining spider community assemblages not only from a taxonomical perspective, but also incorporating information on the organisms’ foraging strategy and body size when monitoring responses to habitat characteristics.

##  Supplemental Information

10.7717/peerj.8856/supp-1 Supplemental Information 1 Dataset that provided the basis for the analyses presented Description of spider data, including a list of spider families sampled, with their guild and the number of individuals collected per family (number of individuals per site and month); morphological measurements taken for each individual (carapace width).

## Additional Information and Declarations

### Field Study Permissions

The following information was supplied relating to field study approvals (i.e., approving body and any reference numbers): Field study was approved by the Vicerrectorado de Investigación de la Universidad de Salamanca (USAL2015/18) and by the owners Francisco Acera, Roberto Díaz, Anibal Leralta, Jesús Carlos Manjón, Manuel Martín, Rafael Morales, Dionisio Moreno, José María Prieto, and Simeón Simón, who allowed us to do the samplings in their orchards.

### Data Availability

The following information was supplied regarding data availability: Data is available in the Supplementary Files.
